# *Plasmodium falciparum* Malaria and Arbovirus Co-Exposure in the Boende Health Zone, Northwestern Democratic Republic of the Congo

**DOI:** 10.3390/tropicalmed11050122

**Published:** 2026-05-05

**Authors:** Solange Milolo Tshilumba, Ynke Larivière, Trésor Zola Matuvanga, Armand Mutwadi, Danoff Engbu, Germain Kapour, Gwen Lemey, Maha Salloum, Maeliss Champagne, Daddy Mangungulu, Pierre Van Damme, Hypolite Muhindo-Mavoko, Vivi Maketa Tevuzula, Joachim Mariën, Martine Peeters, Jean-Pierre Van Geertruyden, Patrick Mitashi-Mulopo

**Affiliations:** 1 Tropical Medicine Department, University of Kinshasa, Kinshasa 01306, Congo; zola.matuvanga@unikin.ac.cd (T.Z.M.); engbudanoff04@gmail.com (D.E.); germainkapour@gmail.com (G.K.); mnglbambi@gmail.com (D.M.); hypolite.muhindo@unikin.ac.cd (H.M.-M.); vivi.maketa@unikin.ac.cd (V.M.T.); 2 Global Health Institute, University of Antwerp, 2610 Antwerp, Belgium; ynke.lariviere@uantwerpen.be (Y.L.); armand.mutwadi@student.uantwerpen.be (A.M.); gwen.lemey@uantwerpen.be (G.L.); maha.salloum@uantwerpen.be (M.S.); jean-pierre.vangeertruyden@uantwerpen.be (J.-P.V.G.); 3 Kinshasa School of Public Health, University of Kinshasa, Kinshasa 01306, Congo; 4 Institut One Health pour l’Afrique, Kinshasa 01306, Congo; 5 Translational Research on HIV and Endemic and Emerging Infectious Diseases (TransVIHMI), National Institute for Health and Medical Research (INSERM), Institute for Research and Sustainable Development (IRD), University of Montpellier, 34394 Montpellier, France; maeliss.champagne@ird.fr (M.C.); martine.peeters@ird.fr (M.P.); 6 Centre for the Evaluation of Vaccination, University of Antwerp, 2000 Antwerp, Belgium; pierre.vandamme@uantwerpen.be; 7 Evolutionary Ecology Group, University of Antwerp, 2610 Antwerp, Belgium; joachim.marien@uantwerpen.be; 8 Virus Ecology Group, Department of Biomedical Sciences, Institute of Tropical Medicine Antwerp, 2000 Antwerp, Belgium

**Keywords:** malaria, arboviruses, co-exposure, seroprevalence, Tshuapa, DRC

## Abstract

**Background:** Malaria remains hyperendemic in the Democratic Republic of the Congo, while arboviral infections are increasingly reported but remain under-surveilled, particularly in remote regions. Overlapping ecological niches and non-specific clinical presentations complicate case management and surveillance. **Methods:** A cross-sectional door-to-door survey was conducted in December 2023 in Inkanamongo village (Lokolia Health Area, Boende Health Zone, Tshuapa Province). Blood samples were collected from 379 adults; malaria infection was assessed by using HRP2-based rapid diagnostic tests, and arboviral IgG antibodies were measured on dried blood spots using Luminex^®^ multiplex immunoassay. Sociodemographic data were collected via standardized questionnaires. **Results:** Malaria prevalence was 51.7% (95%CI: 46.7–56.7). Overall arboviral seroprevalence reached 78.4% (95%CI: 73.1–81.5), dominated by O’nyong-nyong virus, 42.8% (95%CI: 37.6–47.5), Rift Valley fever virus, 32.0% (95%CI: 26.9–36.2), and chikungunya virus, 23.4% (95%CI: 19.0–27.4). Concurrent malaria infection and arboviral exposure were observed in 40.4% (95%CI: 35.6–45.4) of participants. No sociodemographic factors were significantly associated with co-exposure in the multivariable analysis. **Conclusions:** Substantial co-exposure of malaria and multiple arboviruses occurs in this remote Congo Basin setting. Integrated surveillance and improved diagnostics are urgently needed to guide febrile illness management and preparedness in under-resourced regions.

## 1. Introduction

Malaria remains a major endemic disease across the African region, with an estimated 282 million cases and 610,000 deaths reported in Africa region in 2024, and *Plasmodium falciparum* accounts for most of these infections [1]. The Democratic Republic of the Congo (DRC) ranks as the second most affected country in sub-Saharan Africa, contributing over 35 million cases and 12.5% of the global malaria burden, and 11.1% of global malaria deaths. The disease morbidity and mortality are particularly concentrated in rural and remote areas, where healthcare infrastructure is poor, and access to prompt diagnosis and effective treatment is often inadequate [1].

In parallel, arboviral infections, including *dengue virus* (DENV), *chikungunya virus* (CHIKV), *Zika virus* (ZIKV), *yellow fever virus* (YFV), *West Nile virus* (WNV), *Usutu virus* (USUV), *O’nyong-nyong virus* (ONNV), *Rift Valley fever virus* (RVFV), and *Crimean–Congo hemorrhagic fever virus* (CCHFV) are increasingly reported across sub-Saharan Africa [2,3]. These viruses span two main families: Flaviviridae (DENV, ZIKV, YFV, WNV, USUV), and *Togaviridae* (CHIKV, ONNV), with CCHFV classified under *Nairoviridae,* and RVFV under *Phenuiviridae* [2,3]. Arboviral diseases represent a global public health problem, led by dengue, whose incidence varies by year, with an estimated 50–100 million cases annually, including around 500,000 severe cases requiring emergency hospitalization. However, it is important to acknowledge that data from Africa remain limited [4].

Malaria and arboviruses are vector-borne diseases with overlapping ecological niches. Both are transmitted by mosquitoes, including *Aedes spp.* (DENV, CHIKV, ZIKV, YFV); *Culex spp*. (WNV, USUV); and *Anopheles spp*. (ONNV, *Plasmodium ssp*). By contrast, CCHFV is transmitted by *Hyalomma* ticks, with additional zoonotic transmission occurring through contact with infected animal blood or tissues [5]. RVFV has a dual transmission route via mosquitoes (mainly *Aedes* and *Culex*) and direct exposure to infected animal fluids [6]. Overlapping infections with malaria and arboviruses have been documented in several West African countries [7]. However, there is a notable lack of data on co-infection patterns in Central Africa, particularly in the DRC, where environmental conditions are similarly favorable for arbovirus transmission.

Clinically, arboviral infections typically present with non-specific symptoms such as acute fever, headache, myalgia, arthralgia, rash, fatigue, and lymphadenopathy. These symptoms overlap substantially with malaria, making clinical differentiation difficult in endemic settings [7,8,9]. In the absence of reliable laboratory diagnostics, especially in rural settings, arboviral infections are frequently misdiagnosed as malaria, resulting in inappropriate treatment and underestimation of arboviral disease burden [10].

The DRC’s National Malaria Control Program (NMCP) benefits from national-level coordination and widespread deployment of rapid diagnostic tests (RDTs). In contrast, arboviral surveillance remains minimal, particularly in remote provinces such as Tshuapa. Although recent serosurveys have confirmed the circulation of DENV, CHIKV, and WNV in urban centers such as Kinshasa and Matadi [9,11]. Rural provinces remain vastly under-surveyed. Additionally, data on ONNV, USUV, ZIKV, CCHFV, and RVFV remain limited or outdated [12]. This lack of up-to-date seroprevalence and ecological data impedes accurate risk assessment, leaving important gaps in the national arbovirus surveillance framework.

The Tshuapa Province is located within the remote Congo Basin ecosystem. Malaria is endemic in the region and primarily transmitted by *Anopheles gambiae* complex and *Anopheles ziemanni* [13] with 473,000 cases reported in 2024 [14]. Dense forest, high rainfall, and wildlife–human interaction zones offer ideal vector habitats. Given these conditions, most infections are biologically plausible and may be underreported due to diagnostic and surveillance constraints. The frequency and epidemiological characteristics of such infections are still poorly understood.

Recent research underscores the urgent need for integrated disease surveillance systems capable of detecting both malaria and arboviruses within a single diagnostic and reporting framework, particularly in under-resourced regions of sub-Saharan Africa [3]. In areas like Tshuapa Province in the DRC, a region characterized by malaria hyperendemicity, ecological vulnerability, and limited arboviral diagnostics, such platforms could play a crucial role in early outbreak detection, preventive response, and rational case management [10,15].

Although malaria is hyperendemic and ecological conditions favor arboviral transmission, data on the extent of arboviral exposure and its overlap with *Plasmodium* infection in rural Congo Basin settings remain scarce, particularly among asymptomatic populations living in remote forested areas such as Tshuapa Province [12]. Most available studies focus on acute febrile illness and urban or peri-urban settings, leaving a major knowledge gap regarding silent transmission dynamics and co exposure patterns in rural communities [10]. In addition to being a major cause of morbidity and mortality on its own, *Plasmodium* infection may also influence host immune responses to other pathogens. It has been suggested that malaria induced immune activation could modulate and potentially attenuate the clinical manifestations of some arboviral infections, which might contribute to the under-recognition of arboviral disease in endemic areas [4]. This study aims to determine the seroprevalence of selected arboviruses and assess the frequency of co-exposure with *Plasmodium spp* in a rural forest population of Tshuapa Province, DRC. In addition, we explore whether *Plasmodium* infection status is associated with differences in arboviral serological profiles. Arboviral exposure is common in this setting despite limited clinical recognition [7,16]. Moreover, *Plasmodium* infection frequently co-occurs with arboviral exposure and may be associated with altered clinical or immunological overlap [15,17].

## 2. Materials and Methods

### 2.1. Study Setting

**Figure 1:**
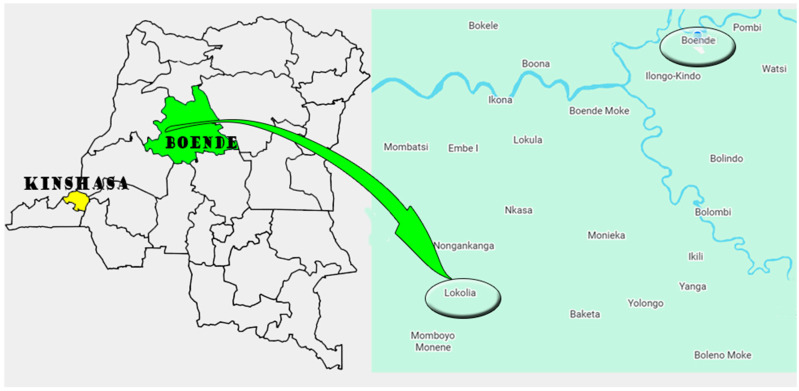
Location of the study site. On the left, a general map of the Democratic Republic of the Congo. On the right, the location of the study site Lokolia Health Area.

The study was conducted in the Boende Health Zone (Figure 1), which is in the Northwestern region of the DRC, specifically in the Tshuapa Province. Spanning approximately 10,775 km^2^, this health zone comprises twelve rural health areas. The area is located in an equatorial environment favorable to mosquito-borne malaria and arboviral transmission. Boende city, the administrative center of the health zone, is a major riverine transport hub. However, access to peripheral areas is often limited by poor road infrastructure and seasonal flooding. Healthcare services in the region are limited, consisting of a network of under-resourced and poorly equipped primary healthcare centers, which impedes both epidemiological surveillance and access to basic healthcare services. Within this zone, the Lokolia health area is notable for its geographical isolation and environmental conditions that facilitate the transmission of vector-borne diseases [18]. Lokolia encompasses several remote rural communities, including the sparsely populated village of Inkanamongo, whose inhabitants primarily engage in subsistence farming, hunting and fishing. Inkanamongo, a village in the Lokolia health zone, was selected for this study because it represents an isolated and poorly monitored environment with ecological conditions favorable to the transmission of malaria and arboviruses.

### 2.2. Study Design and Population

In December 2023, a cross-sectional door-to-door survey was conducted in Inkanamongo village, located within the Lokolia Health Area of the Boende Health Zone, in Tshuapa Province, DRC. The survey aimed to assess arboviral and malaria co-circulation among healthy adults. The sample size was primarily determined by feasibility considerations rather than a formal a priori power calculation. All eligible adults present in Inkanamongo during the survey period were approached, resulting in a convenience sample of 379 participants, which provides reasonable precision for prevalence estimates in this remote, hard-to-reach setting.

### 2.3. Enrollment and Data Collection

All potential participants were approached and provided with a detailed explanation of the study, along with an informed consent form (ICF) available in both French and Lingala. Upon agreeing to participate, individuals signed or placed their fingerprint on two copies of the ICF: one was retained by the investigator, the other was given to the participant.

### 2.4. Sample Collection

Capillary blood samples were collected via fingerprint. A trained laboratory technician or nurse performed the finger prick to collect five blood drops (at least four) from each participant. One drop of approximately 50 µL was used for a malaria rapid diagnostic test (mRDT) for malaria, while the remaining four drops were used to saturate Whatman 3MM filter paper to create dried blood spots (DBSs) for serological analysis. The DBS samples were air-dried at room temperature in an insect-free environment and then stored in ziplocked bags containing approximately 5 cc of silica gel. Subsequently, all DBS samples were preserved at ≤−20 °C until serological testing was performed. A structured and standardized questionnaire was also administered to collect sociodemographic information, including age, gender, marital status, educational level, and household size (i.e., individuals residing under the same roof, sharing meals, and recognizing the authority of a single individual designated as the head of the household).

### 2.5. Laboratory Analysis

Malaria was diagnosed using WHO-prequalified RDTs detecting *Plasmodium falciparum*–specific HRP2 antigen [19]. Capillary blood (~50 µL) was collected via finger prick and applied to test cassettes following the manufacturer’s instructions. Results were interpreted visually within 15–20 minutes. Invalid tests were repeated immediately. Trained staff followed Standard Operating Procedures (SOPs), and quality control included proper storage, expiration checks, and documentation of results. Data was recorded and verified by a second team member.

To assess serological evidence of exposure or infection with emerging arboviruses, this study utilized a multiplex immunoassay approach based on Luminex® technology. This technology was previously validated for serological assessment of emerging arboviruses [12,20]. Antibodies were extracted from DBS using a standardized elution protocol, yielding approximately 5 µL of plasma-equivalent per spot, which was then diluted 1:200 in a buffer containing PBS, 1% BSA, and 0.05% Tween-20. The Bio-Plex 200 system (Luminex®, Bio-Rad, Hercules, CA, USA) was employed to detect IgG antibodies using antigen-coated fluorescent beads, combining the principles of ELISA with flow cytometry. Recombinant antigens from arboviruses were each attached to distinct bead populations. After overnight incubation of eluates with the beads, immune complexes were detected using a biotin-conjugated anti-human IgG antibody and streptavidin–phycoerythrin. The measured signal intensity, expressed as median fluorescence intensity (MFI), was used to estimate the levels of IgG antibodies directed against the various arboviruses (ONNV, WNV, DENV, ZIKV, YFV, CHIKV, RVFV, CCHFV, and USUV) analyzed. For each viral antigen, a positivity threshold was defined based on the mean MFI of negative human controls, increased by three standard deviations, according to the standards used in multiplex immunology. Negative controls were obtained from European donors with no travel to arbovirus-endemic regions and no history of flavivirus vaccination. Samples were confirmed seronegative using IgG/IgM ELISA, immunofluorescence assays, and PCR where applicable, and showed no reactivity across the antigen panel, providing a reliable low-exposure baseline for cut-off determination. This panel of thresholds was used to evaluate the serological response specific to each virus. Any sample with a value above its respective threshold was considered positive. The Luminex® analyses were performed at the laboratory of the International Joint Unit “Transmission, Innovation Pathways, Infectious Diseases” (TransVIHMI), affiliated with IRD (UMI 233), INSERM (U1175), and the University of Montpellier.

### 2.6. Statistical Analysis

Data were cleaned and analysed using the R statistical environment (R studio version 2025.09.2 Build 418). Initial data quality checks included inspection of variable structure, distributions, and completeness. Age was categorized into three groups (18–49, 50–64, and ≥65 years). Household size was grouped as 1–3, 4–6, and ≥7 members. Gender, age group, marital status, educational level, and household size were treated as categorical variables in all analyses. Laboratory results for malaria and arboviruses were recorded as binary outcomes (negative/positive). For arboviruses, individual test results were harmonized to binary indicators. A composite variable (“any arbovirus”) was created to indicate serological evidence of exposure to at least one arboviral (IgG positivity). Co-exposure at the individual level was operationally defined as malaria infection detected by HRP2 RDT together with arboviral exposure (positivity for at least one arboviral IgG).

Descriptive analyses were conducted to summarize the demographic characteristics of the study population. Proportions and corresponding 95% confidence intervals (CIs) were calculated for each demographic variable using the Wilson method. The prevalence of malaria was estimated overall and stratified by demographic characteristics. Similarly, the seroprevalence against any arbovirus infection was calculated overall and by demographic subgroup. For arboviral infections, overall prevalence and 95% CIs were estimated for each virus. Graphical summaries, including bar charts with error bars, were used to visualize seroprevalences across demographic groups and pathogens. Co-circulation between active malaria infection and arboviruses exposure was assessed by estimating the overall prevalence of co-circulation and its 95% CI, followed by stratified analyses according to demographic variables. Cross-tabulations between malaria and each arbovirus were generated to describe joint distributions and co-occurrence patterns.

To explore and visualize patterns of single and multiple infections co-occurrence, we used combinatory analysis and constructed a Venn diagram using the *ggVennDiagram* package in R. We defined nine disease categories based on laboratory confirmation: malaria (positive rapid diagnostic test), ONNV, DENV (positive NS1 antigen test across four serotypes), CHIKV, ZIKV, USUV, WNV, RVF, and CCHFV. For diseases with multiple diagnostic tests (DENV, WNV, RVFV, CCHFV), cases were considered positive if any of the specified tests returned a positive result.

To identify factors associated with the co–circulation between malaria infection and arbovirus exposure, logistic regression analyses were performed. Univariate logistic regression models were first fitted to assess the association between each demographic variable and co-exposure status. Finally, a multivariable explicative logistic regression model was constructed that included all demographic variables simultaneously to account for potential confounders. Model results were reported as regression coefficients with corresponding standard errors, test statistics, and p-values. Statistical significance was assessed using a two-sided alpha level of 0.05.

### 2.7. Ethical and Administrative Clearance

Prior to enrollment, the study’s objectives, potential benefits, and associated risks were thoroughly explained to all participants in either French or Lingala. Written informed consent was obtained from each participant. Ethical approval for this study was granted by the National Health Ethics Committee of the DRC (authorization reference: 375/CNES/BN/PMMF/2022). Administrative authorization was also obtained from the Head of the Tshuapa Provincial Health Division.

## 3. Results

The study involved 379 participants, of which 156 were women and 223 were men (Table 1). Most of the participants were married or living in common-law relationships. The median age was 41.0 years (IQR:33–50), with a range of 18 to 76. Educational backgrounds varied, but a large portion, 62.8% (CI95%: 57.8-67.5), had never received formal education. The median household size was 6 (IQR: 4–8) people.

**Figure 2:**
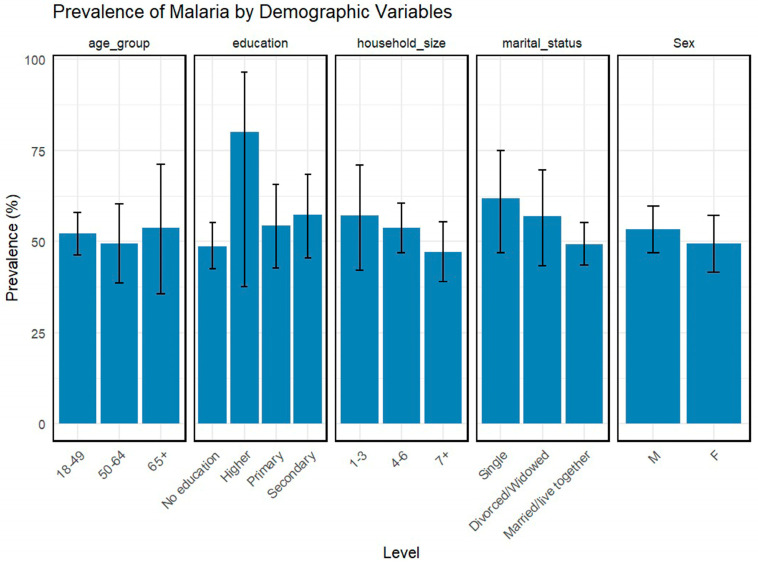
Prevalence of asymptomatic malaria infection according to sociodemographic subgroups.

Malaria was most often detected as a single infection, accounting for 51.7% of cases. Prevalence varied notably across sociodemographic subgroups (Figure 2), revealing complex and sometimes unexpected patterns of risk. By age, malaria prevalence was 52.8% (CI95%: 46.3–58.0) in the 18–49 age group, 50.2% (95% CI: 38.5–60.3) among individuals aged 50–64 years, and 55.4% (95% CI: 35.5–71.2) in those aged 65 and older. Marital status showed a distinct association: single individuals exhibited the highest prevalence 61.4% (95%CI: 46.8–75.0), while those married or cohabiting had the lowest 49.3% (95% CI: 43.6–55.1). Prevalence was substantial among individuals with higher education 81.2%, (95% CI: 37.6–96.4), compared to those with secondary 56.4%(95% CI: 45.5–68.4), primary 53.1% (95% CI: 42.7–65.7), or no formal education 48.6%(95%CI: 42.5–55.1). Confidence intervals indicated considerable variability, particularly within the highly educated subgroup. Malaria prevalence was 55.3% in men (95% CI: 46.8–59.8) and 49.7% in women (95% CI: 41.6–57.1), and the difference was not statistically significant. Household size appeared inversely related to malaria prevalence: smaller households showed higher rates (56.8%), which decreased with increasing household size (53.4% for households with 4–6 members and 46.1% for those with 7 or more).

**Figure 3:**
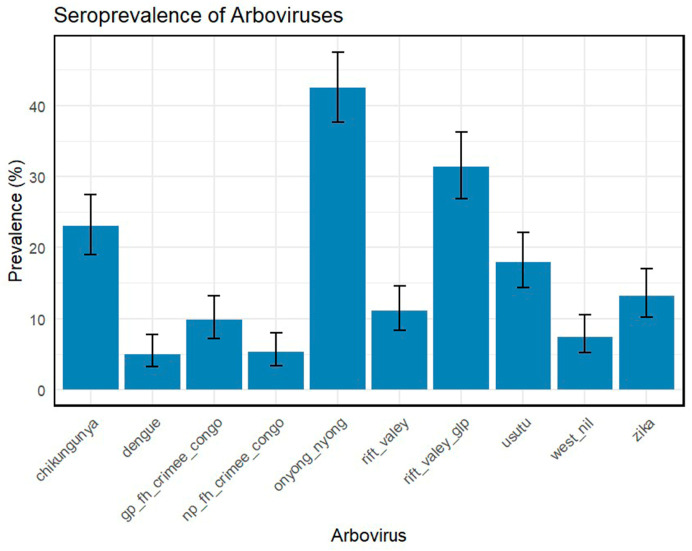
Seroprevalence of arboviruses among participants.

The seroprevalence estimates indicated the presence of several arboviruses in the population. The highest seroprevalence was observed for ONNV, detected in 42.8% of participants (95% CI: 38.1–47.3). RVFV glycoprotein followed at 32.0% (95% CI: 27.8–36.5), and CHIKV at 23.4% (95% CI: 19.3–27.8). Moderate prevalence was seen for USUV (17.9%, 95% CI: 14.2–22.2), ZIKV (13.6%, 95%CI: 10.4–17.5), RVFV (11.2%, 95% CI: 8.3–14.6), CCHFV glycoprotein (gp_IFN-CCHFV; 9.8%, 95% CI: 7.1–13.2), and nucleoprotein (np_IFN-CCHFV; 5.7%, 95% CI: 3.7–8.5). Antibodies against DENV were detected at lower levels 5% (95% CI: 3.2–7.7). WNV also exhibited a low prevalence, ranging from 0.6% based on envelope protein (95% CI: 0.2–1.9) to 6.8% overall (95% CI: 4.6–9.7). These results show co-exposure to multiple arboviruses, with ONNV predominating (Figure 3).

**Figure 4:**
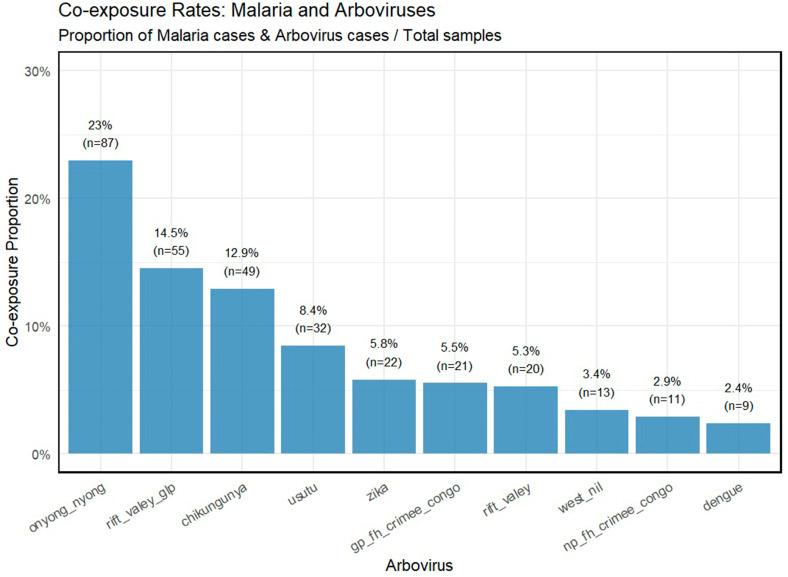
Seroprevalence of malaria–arbovirus co-exposures according to sociodemographic characteristics.

The seroprevalence of co-exposure with malaria and arboviruses varied across sociodemographic groups (Figure 4). Among age categories, the highest prevalence was observed in individuals aged 18–49 years (41.7%), followed by those aged 50–64 years (37.7%) and individuals aged 65 years and older (34.6%). With respect to marital status, single individuals exhibited the highest prevalence (52.4%), while those divorced or widowed had a prevalence of 41.2%. The lowest prevalence was reported among individuals who were married or living together (38.5%). By education level, co-exposure prevalence was highest among individuals with higher education (80.0%), followed by those with secondary education (51.5%), primary education (41.2%), and no formal education (36.1%). The widest confidence intervals were observed in the higher education group, indicating greater variability in this category. Participants who were men had a slightly higher prevalence (42.6%) compared to women (37.2%). Regarding household size, individuals living in households with 1–3 members had the highest prevalence (40.5%), followed by those in households with 4–6 members (41.8%). The lowest seroprevalence was observed in households with seven or more members (38.2%). No independent risk factors may have been found in this research based on multivariable analysis. However, univariable analyses may be important for prediction in clinical epidemiology (i.e., predicting a higher risk for either any arboviral infection or malaria infection (Table 2)).

**Figure 5:**
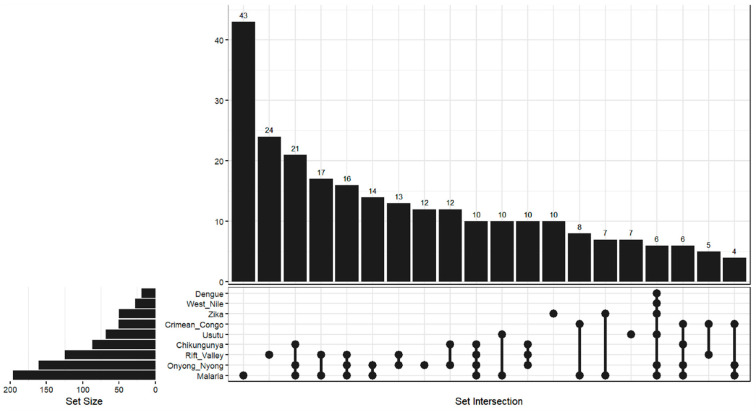
The UpSet plot shows how often single or combined arboviruses seropositivity patterns appear together with malaria, and how much they overlap. The left panel shows the overall number of cases per pathogen, irrespective of co-exposure. The upper bar chart shows the number of participants with each serological combination, while the dot matrix indicates the viruses and parasite included in each pattern. The figure shows how different arboviruses and malaria are exposed to each other.

Co-exposure of multiple arboviruses and malaria was detected. Double positivity was observed in 86 samples (86/379; 22.7%), triple positivity was observed in 51 samples (51/379; 13.5%), and at least quadruple positivity was observed in 22 samples (22/379; 5.8%). Malaria/RVFV (17/379), Malaria/ONNV (14/379), Malaria/USUV (10/379), Malaria/CCHFV (8/379), Malaria/ZIKV (7/379), ONNV/RVFV (13/379), ONNV/CHIKV (12/379), Malaria/ONNV/CHIKV (21/379), Malaria/ONNV/RVFV (16/379), Malaria/ONNV/CHIKV/RVFV (10/379), Malaria/ONNV/CHIKV/CCHFV (6/379), Malaria/ONNV/CCHFV (4/379), Malaria/ONNV/USUV/ZIKV/WNV/DENV (6/379) (Figure 5).

## 4. Discussion

This study provides novel evidence of malaria infection in the context of substantial arbovirus co-infection in the reflected by high IgG seroprevalence, in Tshuapa Province, DRC, a remote, forested region with hyperendemic malaria transmission and minimal arboviral surveillance infrastructure. The notably high malaria prevalence (51.7%), combined with a remarkably high overall arboviral seroprevalence (78.4%) and a substantial co-exposure rate (23%) among individuals seropositive for at least one arbovirus, underscores the complex epidemiological landscape of febrile illness in tropical, vector-rich environments. These findings align with previous research from other African regions demonstrating co-circulation of Anopheles and Aedes mosquitoes and overlapping transmission cycles for multiple vector-borne pathogens [7,21].

The observed malaria prevalence of 51.7% among asymptomatic adults highlights the presence of a substantial parasite reservoir, posing a substantial challenge to elimination strategies. Sustainable malaria control will require the inclusion of asymptomatic reservoir treatment as a central pillar of elimination efforts. Studies have shown that asymptomatic carriers can sustain transmission even in low-endemic settings, making them a critical target for intervention [22,23,24].

In the Congo Basin, co-exposure to arboviruses and malaria may be sustained by interacting environmental, ecological, and human factors. Seasonal rainfall, high temperatures, stagnant water, vector adaptability, population mobility, and routine activities near mosquito habitats may increase mosquito abundance and human–vector contact, thereby facilitating arbovirus transmission in ecosystems that also support malaria transmission [25]. However, high seroprevalence may reflect past infection, reinfection, ongoing transmission, or increased exposure associated with socioeconomic activities near mosquito breeding sites. The overlap between arbovirus seropositivity and malaria infection could suggest shared exposure risks and the potential co-circulation of vector-borne pathogens, underscoring the need to strengthen differential diagnosis in malaria-endemic settings.

ONNV was the most frequently detected arbovirus among individuals with concurrent malaria infection and arboviral exposure (15.3%). It also exhibited the highest IgG seroprevalence in mono-infections (42.5%) consistent with its transmission by *Anopheles* mosquitoes, the same *genus* responsible for malaria which are highly prevalent in the region [13]. This ecological overlap plausibly facilitates simultaneous exposure and co-circulation.

The substantial seroprevalence of RVFV (32.0% glycoprotein, 11.2% nucleoprotein), and CCHFV (9.8% glycoprotein, 5.7% nucleoprotein) may indicate low-level endemic transmission or recurrent zoonotic exposure in peridomestic and sylvatic settings. Unlike other African regions where livestock play a central role, especially in RVFV transmission, the scarcity of domestic animals in surveyed rural areas points to a possible role of wild fauna and forest vectors [6,26]. Given homogeneity in participant lifestyles, behavioral differences are unlikely to explain regional variation in CCHFV, and RVFV exposure. While these findings are consistent with arbovirus exposure at the forest–agriculture interface, caution is warranted due to the risk of serological cross-reactivity, especially in malaria-endemic populations. However, serological cross-reactivity, especially from exposure to common pathogens, may compromise results in specificity in malaria-endemic areas [27,28]. Broader, standardized sampling, and improved diagnostic specificity are essential for accurately assessing transmission dynamics and infection risk.

Despite broader documentation of CHIKV across the DRC, ONNV seroprevalence in this cohort surpassed that of CHIKV. Given the antigenic and phylogenetic similarity between the two alphaviruses, serological misclassification is plausible in the absence of confirmatory neutralization or molecular diagnostics. This raises the possibility that ONNV is under-recognized in Central Africa and may contribute significantly to undiagnosed febrile illness [29].

Other detected flaviviruses, USUV (17.9%), ZIKV (13.6%), and DENV (5%), showed lower-level circulation, indicating likely endemic but silent or intermittent transmission patterns within forested zones harboring competent vectors. The detection of multiple flaviviruses, including the WNV, up to 6.8%, supports the notion of cumulative exposure and raises concerns over potential antibody cross-reactivity, a known challenge in flavivirus serology [30,31].

Sociodemographic stratification revealed that adults aged 18–49 years, those with higher education levels, and unmarried individuals had substantial co-exposure rates, although these differences were not statistically significant. These patterns could probably reflect differences in occupational or environmental exposure. The substantial seroprevalence of co-exposure in both men and women may reflect similar exposure risks in the study site. Women may be exposed during water-related and domestic activities, whereas men may be exposed through hunting, fishing, gardening, and fieldwork. These activities may result in comparable exposure to *Aedes* and *Anopheles* mosquito bites. However, such interpretations remain cautious and need further data. In contrast, lower co-exposure rates in households with more members may suggest protective behavioral norms such as collective use of insecticide-treated bed nets, though further investigation is needed to explore this hypothesis.

These findings emphasize the significant deficiencies in the DRC’s ability to detect and respond to emerging vector-borne diseases. The high prevalence of arboviruses and malaria in regions such as Tshuapa, where diagnostic resources are limited, suggests that transmission is ongoing and silent. The presence of asymptomatic malaria and arbovirus carriers further complicates surveillance and control efforts, enabling pathogens to spread undetected. With few vaccines available, the DRC’s surveillance program must prioritize vector monitoring and control. Given the high risk of emergence and amplification, regional coordination on surveillance, diagnostics, and preparedness is essential. A fragmented national-level approach is unlikely to suffice. Moving forward, a comprehensive “One Health” strategy that integrates human, animal, vector and environmental health is crucial for effective disease prevention.

This study has some limitations that affect the interpretation of the results. The most significant of these is serological cross-reactivity among flaviviruses [30,31], which can lead to an overestimation of prior exposure in the absence of confirmatory assays like neutralization. While serology remains essential for arboviral surveillance, these findings emphasize the urgent need for more discriminating diagnostic tools that can reliably differentiate closely related viruses. Furthermore, the absence of molecular diagnostics and IgM tests means that detection is confined to past infections. The use of histidine-tagged recombinant antigens may have introduced false positives due to antibody cross-reactivity in populations with a high prevalence of malaria [32]. Additionally, key behavioral and environmental risk factors such as the use of bed nets, occupational exposure, or other factors influencing individual vulnerability to arboviral infections were not assessed thereby limiting the depth of insight into transmission dynamics. Lastly, the clinical management of febrile patients in this region remains poorly characterized and warrants further investigation.

Nonetheless, the findings highlight the urgent need for integrated, cross-pathogen surveillance strategies in tropical ecosystems where parasitic and viral vector-borne pathogens co-circulate. Diagnostic refinement, particularly using tag-free recombinant antigens, multiplex serology, and nucleic acid-based confirmation will be critical to improving the accuracy of burden estimates, detecting emerging threats, and guiding evidence-based clinical management of febrile illness in resource-limited settings.

## Figures and Tables

**Table 1:** Sociodemographic characteristics of study participants.

Variables	Level	N	Total	Prop	CI95%: Lower	CI95%: Upper
Gender	W	118	156	75.6	68.3	81.7
	M	176	223	78.9	73.1	83.8
Age__group	18–49	219	276	79.3	74.2	83.7
	50–64	56	77	72.7	61.9	81.4
	65+	19	26	73.1	53.9	86.3
Marital status	Divorced/Widowed	41	51	80.4	67.5	89
	Married/live together	219	286	76.6	71.3	81.1
	Single	34	42	81	66.7	90
Study level	Higher	5	5	100	56.6	100
	No education	176	238	73.9	68	79.1
	Primary	54	68	79.4	68.4	87.3
	Secondary	59	68	86.8	76.7	92.9
Housekeeping group	1–3	31	42	73.8	58.9	84.7
	4–6	157	201	78.1	71.9	83.3
	7+	106	136	77.9	70.3	84.1

**Table 2:** Sociodemographic factors predicting Malaria and Arboviruses co-exposure in the Boende Health Zone, December 2023.

Demographic Characteristics	Univariate Analysis		Multivariate Analysis	
	OR (CI 95%)	*p*-Value	aOR (CI 95%)	*p*-Value
(Intercept)			0.83 (0.34–2.02)	0.69
Gender				
Men	Ref		Ref	
Women	0.80 (0.52–1.21)	0.29	0.92 (0.44–1.93)	0.83
Age Group				
18–49 years	Ref		Ref	
50–64 years	0.85 (0.5–1.41)	0.53	0.87 (0.37–2.19)	0.75
65+ years	0.74 (0.31–1.69)	0.49	0.4 (0.11–1.6)	0.18
Marital Status				
Single	Ref		Ref	
Separated/widow(ed)	0.64 (0.28–1.45)	0.28	0.78 (0.17–3.32)	0.74
Married/live together	0.57 (0.29–1.09)	0.09	0.73 (0.19–2.26)	0.61
Education				
No education	Ref		Ref	
Higher	7.03 (1.09–139.8)	0.08	13.86 (1.16–173.0)	0.99
Primary	1.24 (0.71–2.14)	0.45	0.96 (0.39–2.47)	0.92
Secondary	1.87 (1.09–3.24)	0.02*	2.77 (0.94–10.25)	0.09
Household Size				
1 to 3	Ref		Ref	
4 to 6	1.06 (0.54–2.11)	0.88	1.51 (0.5–4.24)	0.44
7+	0.91 (0.45–1.87)	0.79	1.67 (0.51–5.2)	0.38

significant *p*-value (<0.05).

## Data Availability

The authors declare that the relevant data generated in this study are included in the article, and additional supporting data are available from the corresponding author upon reasonable request.

## Abbreviations

CCHFV	Crimean–Congo hemorrhagic fever virus
CHIKV	Chikungunya virus
DENV	Dengue virus
ONN	O’nyong-nyong virus
RVFV	Rift Valley fever virus
USUV	Usutu virus
WNV	West Nile virus
YFV	yellow fever virus
ZIKV	Zika virus
DRC	Democratic Republic of the Congo
